# On-Pump vs Off-Pump coronary artery bypass surgery in atrial fibrillation. Analysis from the polish national registry of cardiac surgery procedures (KROK)

**DOI:** 10.1371/journal.pone.0231950

**Published:** 2020-04-22

**Authors:** Mariusz Kowalewski, Marek Jasiński, Jakub Staromłyński, Marian Zembala, Kazimierz Widenka, Mirosław Brykczyński, Jacek Skiba, Michał Zembala, Krzysztof Bartuś, Tomasz Hirnle, Inga Dziembowska, Piotr Knapik, Zdzisław Tobota, Bohdan Maruszewski, Piotr Suwalski, on behalf of KROK Investigators

**Affiliations:** 1 Department of Cardiac Surgery, Central Clinical Hospital of the Ministry of Interior, Centre of Postgraduate Medical Education, Warsaw, Poland; 2 Thoracic Research Centre, Collegium Medicum, Nicolaus Copernicus University, Innovative Medical Forum, Bydgoszcz, Poland; 3 Cardio-Thoracic Surgery Department, Heart and Vascular Centre, Maastricht University Medical Centre, Maastricht, the Netherlands; 4 Department and Clinic of Cardiac Surgery, Wroclaw Medical University, Wroclaw, Poland; 5 Department of Cardiac, School of Medicine with the Division of Dentistry in Zabrze, Vascular and Endovascular Surgery and Transplantology, Medical University of Silesia in Katowice, Zabrze, Poland; 6 Division of Cardiac Surgery, Heart and Lung Transplantation and Mechanical Circulatory Support, Silesian Center for Heart Disease, Zabrze, Poland; 7 Clinical Department of Cardiac Surgery, District Hospital no. 2, University of Rzeszów, Poland; 8 Department of Cardiac Surgery, Pomeranian Medical University, Szczecin, Poland; 9 Department of Cardiac Surgery, 4 Military Clinical Hospital Centre for Heart Diseases, Wroclaw, Poland; 10 Department of Cardiovascular Surgery and Transplantology, Jagiellonian University, John Paul II Hospital, Krakow, Poland; 11 Department of Cardiosurgery, Medical University of Bialystok, Bialystok, Poland; 12 Department of Pathophysiology, Faculty of Pharmacy, Collegium Medicum, Nicolaus Copernicus University, Toruń, Poland; 13 Department of Anaesthesiology, Intensive Therapy and Emergency Medicine, Silesian Centre for Heart Diseases, Medical University of Silesia, Zabrze, Poland; 14 Department of Pediatric Cardiothoracic Surgery, The Children's Memorial Health Institute, Warsaw, Poland; Scuola Superiore Sant'Anna, ITALY

## Abstract

**Background:**

No single randomized study has ever before addressed the safety of On-Pump coronary artery bypass grafting (CABG) vs Off-Pump CABG in the setting of atrial fibrillation (AF) and data from small observational samples remain inconclusive.

**Methods and findings:**

Procedural data from KROK (Polish National Registry of Cardiac Surgery Procedures) were retrospectively collected. Of initial 188,972 patients undergoing CABG, 7,913 presented with baseline AF (76.0% men, mean age 69.1±8.2) and underwent CABG without concomitant valve surgery between 2006–2019 in 37 reference centers across Poland. Mean follow-up was 4.7±3.5 years (median 4.3 IQR 1.7–7.4). Cox proportional hazards models were used for computations. Of included patients, 3,681 underwent On-Pump- (46.52%) as compared to 4,232 (53.48%) who underwent Off-Pump CABG. Patients in the latter group less frequently were candidates for complete revascularization (P<0.001). In an unadjusted comparison, On-Pump surgery was associated with significantly worse survival at 30 days: HR: 1.28; 95%CIs: (1.07–1.53); P = 0.007. Along the 13-year study period, the trend shifted in favor of On-Pump CABG: HR: 0.92; 95%CIs: (0.83–0.99); P = 0.005. After rigorous propensity matching, 636 pairs were identified. The direction and magnitude of treatment effects was sustained with HRs of 3.58; (95%CIs: 1.34–9.61); p = 0.001 and 0.74; [95%CIs: 0.56–0.98]; p = 0.036) for 30-day and late mortality respectively.

**Conclusions:**

Off-Pump CABG offered 30-day survival benefit to patients undergoing CABG surgery and presenting with underlying AF. On-Pump CABG was associated with significantly improved survival at long term.

## Introduction

Although the presence of atrial fibrillation (AF) in patients undergoing coronary artery bypass grafting (CABG) is much less than their mitral valve surgery counterparts, still approximately 6% of patients presenting for coronary surgical procedures have preoperative AF [1–3] that often plays as a marker for high-risk patients’ populations [4]. This percentage is known to further increase with older age, and depressed left ventricular function [5] that is in patients more and more frequently referred for CABG surgery. There exists robust evidence to support performing CABG in higher risk patients [6,7] without the use of cardiopulmonary bypass (CPB) (Off-Pump CABG) to avoid deleterious effects of extracorporeal circulation (ECC) and its consequences: transfusions, renal failure, bleeding and cerebrovascular events [8]. On the other hand, On-Pump CABG and arrested heart offer bloodless operative field and allow complete revascularization in most, often very complex cases [9–11].

No single randomized study has ever before addressed the safety of On-Pump CABG vs Off-Pump CABG in this particular setting of AF and data from small observational samples remains inconclusive [12,13]. Driven by this fact, we designed an analysis, in which we report long-term survival results after On-Pump and Off-Pump CABG in AF from the Polish National Registry of Cardiac Surgery Procedures (Krajowy Rejestr Operacji Kardiochirurgicznych [KROK]).

## Methods

### Registry design

The current work represents an anonymous registry analysis; the long-term data are provided by the Polish NHS; IRB approval was lifted. All data were collective in a retrospective fashion from the KROK registry (available at: www.krok.csioz.gov.pl). The registry is an ongoing, nationwide, multi-institutional registry of heart surgery procedures in Poland [14]. The registry is an initiative of the Society of Polish Cardiac Surgeons in cooperation with the Polish Ministry of Health that commenced in 2006 and collects data from all 37 heart surgery centers in Poland (List of contributing centers—Appendix). Centers enrolling patients in the KROK registry are required to transfer the data concerning every cardiac surgery to the central database in the National Centre for Healthcare Information Systems at the Ministry of Health and are financially liable for data integrity and completeness. Follow-up data regarding mortality were obtained from the National Health Fund—the nationwide, obligatory, public health insurance institution in Poland and incorporated to the registry. A registry module for collecting the data regarding myocardial infarctions (MI), hospitalizations due to unstable angina, subsequent revascularizations, strokes and other complications was under construction at the time of analysis.

### Data collection

A detailed questionnaire, defined according to standard definitions, including demographic data, history, physical findings, management, imaging studies, and outcomes, was developed. Data were collected either at presentation or by physician review of the hospital records and were forwarded to the KROK registry. The forms were reviewed for clinical face- and analytical internal -validity.

### Study population and clinical variables

Using the KROK participant user file, we identified adult patients undergoing CABG surgery between 2006–2019. Excluded were those without history of AF or AF at time of presentation and CABG procedures combined with valve(s) surgery. No further exclusion criteria were imposed with regard to patients’ baseline status. For patients undergoing CABG surgery, we considered and report 3 categories of variables as potentially influencing the primary endpoint: 1) baseline demographics: age, gender, EuroSCORE [15], diabetes, body mass, hypertension, poor mobility, pulmonary hypertension, chronic kidney disease, vascular disease, chronic lung disease and LVEF; 2) extent of CAD: previous MI, previous PCI (percutaneous coronary intervention), left main (LM) disease; and 3) surgical characteristics: redo-surgery, endocarditis, cardiogenic shock, intra-aortic balloon pump (IABP), critical preoperative state, iv. inotropes/nitrates, aortic no-touch, total arterial revascularization [TAR]) and completeness of revascularization.

Primary endpoint assessed was late survival in On-Pump vs Off-Pump CABG. Analyses of early postoperative mortality (<24 hours) and 30-day mortality were performed as well. In-hospital complications as well as length of intensive care unit (ICU) and hospital stay (HLoS) are reported.

### Statistical analysis

Missing data were handled with artificial neural networks using Long Short-Term Memory hidden units [16, 17] only to the threshold of up to 5% of missing data [18]. Continuous, normally distributed variables were summarized as mean±standard deviation; variables with non-normal distributions were summarized as median (interquartile range; IQR) and compared with the Mann–Whitney U test or standard t test as appropriate. Categorical variables were expressed as number (%) and compared with the Fisher exact test. Cox proportional-hazards models were used to determine factors related the event-free survival. The ensuing statistical models were used to define the Hazard Ratios (HRs) point estimate and 95% confidence intervals (95% CI) of the effect size and to evaluate the differences with respect to mode of CABG surgery. Respective HRs for the comparison On-Pump vs Off-Pump CABG were calculated and reported first for the univariable Cox proportional-hazards model taking into account all sets of variables categorized by: 1) baseline demographics; 2) extent of CAD; and 3) surgical characteristics. Next, a multivariable model was built, again stratified on the 3 sets of variables. Interaction between univariable and multivariable was assessed with the use of the Cochran–Mantel–Haenszel test after data were stratified. Multivariable model was then tested for multicollinearity.

Propensity score analysis was performed to balance possible confounding between the two study groups with regard to selected variables in order to prevent any bias related to the initial selection of patients for CABG surgery. Propensity scores were computed using a multiple logistic regression model, in which the dependent variable was concomitant ablation and the independent variables were the ones for which the given variable returned an estimated effect of ≥0.1 change in respective HR after multiple logistic regression. Regression adjustment was then fitted resulting in increased precision for continuous outcome as described by Steyerberg [19]. In the final model, we tested the zero variance using the model proposed by Drikwandi et al. [20] to obtain good operating characteristics with respect to type I error and power. A greedy match using nearest-neighbor method was used and 1-to-1 ratio, without replacement, within a specific caliper width of 0.2 SD of the LOGIT of the estimated propensity score. Propensity scores along with Wald (χ^2^) are reported with corresponding 95%CIs. To verify the balance between On-Pump vs Off-Pump CABG groups after PS-matching, the standardized mean differences (SMDs) were computed. For the selected PS-matched population univariable and multivariable Cox proportional hazard models were tested again and statistical differences reported. Overall late and 30-day mortality was assessed with Kaplan Meier curves fitted before (unadjusted model) and after PS-matching. As a sensitivity analysis to assess the survival after On-Pump vs Off-Pump CABG, patients were stratified according to defined subgroups and respective models unadjusted and PS-matched redone. STATA MP v13.0 software (StataCorp, College Station, TX) was used for computations.

## Results

**Fig 1:**
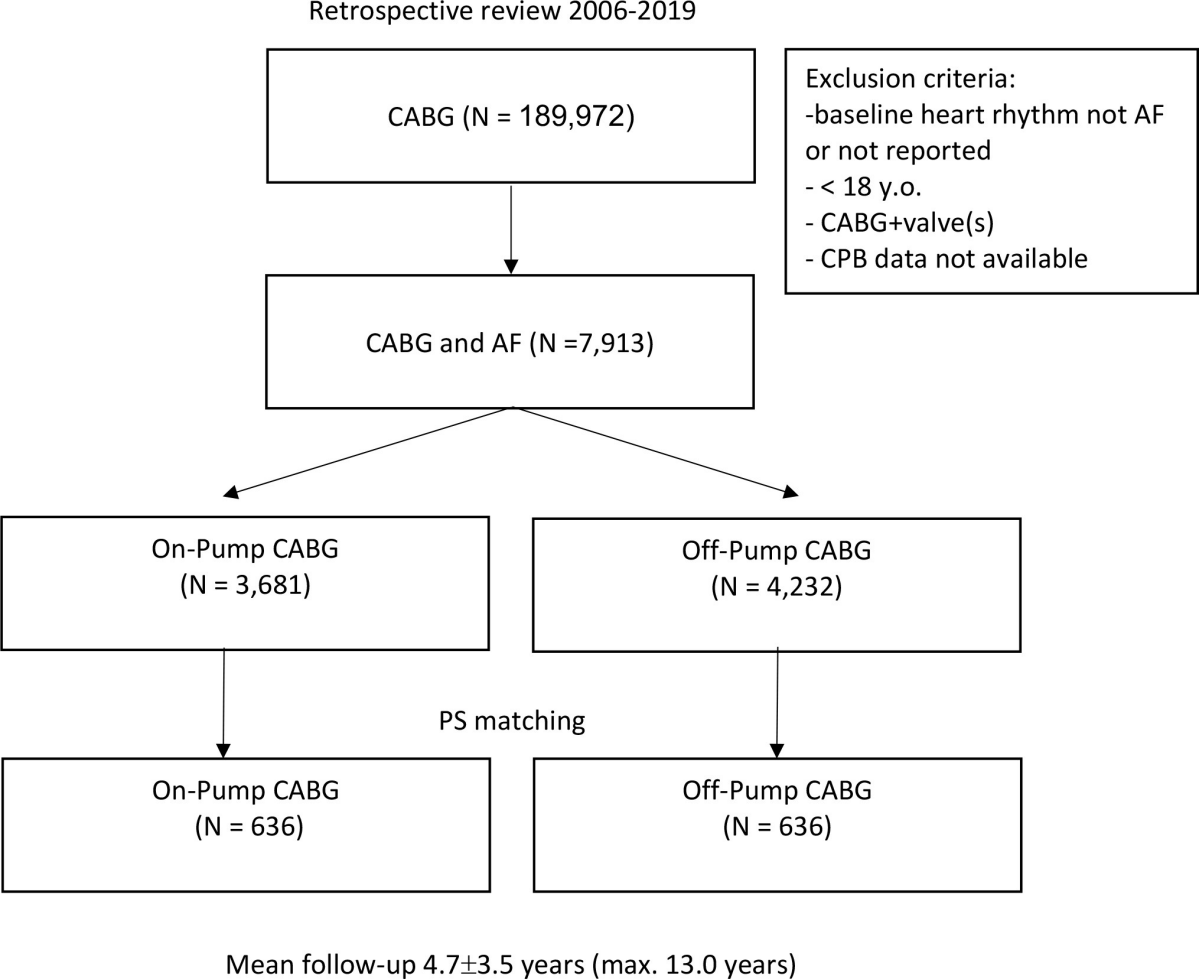
Flow diagram of the current study cohort undergoing On-Pump and Off-Pump CABG surgery in concomitant atrial fibrillation. AF, atrial fibrillation; CABG, coronary artery bypass grafting; PS, propensity score.

During 13-year study period 188,972 patients undergoing CABG surgery were identified. Among those, 7,913 initially presented with AF and did not undergo concomitant valvular surgery. Subjects were divided into On-Pump- (3,681 [46.52%]) and Off-Pump CABG (4,232 [53.48%]). Fig 1. Mean follow-up was 4.7±3.5 years (median 4.3 IQR 1.7–7.4).

### Baseline characteristics

Baseline characteristics along with clinical and surgical data are listed in Table 1. There were no marked differences between On-Pump and Off-Pump CABG with respect to age and distribution of patients across baseline LVEF percentages. Subjects undergoing Off-Pump CABG were more often female (p<0.001) and diabetic (p<0.001) yet lower risk (eg. EuroSCORE <2: 71% vs 65%; p<0.001) as compared to On-Pump CABG. On-Pump CABG group included more three-vessel disease patients (p<0.001) that however less frequently had history of MI (p<0.001)

**Table 1:** Preoperative characteristics before and after PS-matching.

Variable		All patients				PS-matched patients			
		Total (7,913)	On-Pump CABG (3,681)	Off-Pump CABG (4,232)	P_value_	Total (1,272)	On-Pump CABG (636)	Off-Pump CABG (636)	P_value_
***Baseline characteristics***									
age years (median [IQR])		70 [63–75]	70 [63–75]	70 [64–75]	0.476	68 [61–74]	68 [61–73]	68 [61–74]	0.788
	<50	121 (1.53%)	49 (1.33%)	72 (1.70%)	0.181	19 (1.49%)	10 (1.57%)	9 (1.42%)	0.879
	50–70	3,751 (47.40%)	1,761 (47.84%)	1,990 (47.02%)	0.468	697 (54.79%)	349 (54.87%)	348 (54.72%)	0.913
	>70	4,041 (51.07%)	1,871 (50.83%)	2,170 (51.28%)	0.691	556 (43.71%)	277 (43.55%)	279 (43.87%)	0.924
gender									
	male	6,016 (76.03%)	2,883 (78.32%)	3,133 (74.03%)	<0.001	1,019 (80.11%)	508 (79.87%)	511 (80.35%)	0.833
	female	1,897 (23.97%)	798 (21.68%)	1,099 (25.97%)		253 (19.89%)	128 (20.13%)	125 (19.65%)	
EuroSCORE (median [IQR])		1.35 [0.89–2.43]	1.49 [0.98–2.69]	1.22 [0.82–2.23]	<0.001	0.97 (0.74–1.33)	0.97 [0.74–1.32]	0.96 [0.75–1.34]	0.874
	<2	5,378 (67.96%)	2,375 (64.52%)	3,003 (70.96%)	<0.001	1,114 (87.58%)	558 (987.73%)	556 (87.42%)	0.865
	2–5	1,780 (22.49%)	888 (24.12%)	892 (21.08%)	0.001	152 (11.95%)	75 (11.79%)	77 (12.10%)	0.895
	>5	755 (9.54%)	418 (11.36%)	337 (7.96%)	<0.001	6 (0.47%)	3 (0.47%)	3 (0.47%)	0.999
diabetes		3,262 (41.22%)	1,455 (39.53%)	1,807 (42.70%)	0.004	483 (37.97%)	241 (37.89%)	242 (38.05%)	0.954
	diet only	489 (6.18%)	160 (4.35%)	329 (7.77%)	<0.001	52 (4.09%)	26 (4.09%)	26 (4.09%)	0.999
	oral hypoglycemic drugs	1,401 (17.71%)	712 (19.34%)	689 (16.28%)	<0.001	241 (18.94%)	127 (19.97%)	114 (17.425)	0.352
	Insulin ± oral hypoglycemic drugs	1,374 (17.36%)	586 (15.92%)	788 (18.62%)	0.002	185 (14.54%)	86 (13.52%)	99 (15.57%)	0.301
smoking		4,787 (60.50%)	2,074 (56.34%)	2,713 (64.11%)	<0.001	161 (12.66%)	75 (11.79%)	86 (13.52%)	0.354
hypertension		7,136 (90.18%)	3,331 (90.49%)	3,805 (89.91%)	0.368	1,138 (89.47%)	570 (89.62%)	568 (89.31%)	0.855
hyperlipidemia		5,024 (63.49%)	2,229 (60.55%)	2,795 (66.04%)	<0.001	803 (63.12%)	398 (62.58%)	405 (63.68%)	0.684
poor mobility		401 (5.07%)	224 (6.09%)	177 (4.18%)	<0.001	25 (1.97%)	11 (1.73%)	14 (2.20%)	0.544
BMI (median [IQR])		28.4 [25.7–31.5]	28.5 [25.8–31.6]	28.4 [25.6–31.5]	0.273	28.6 [26.1–31.4]	28.6 [26.2–31.5]	28.7 [26.0–31.6]	0.377
pulmonary hypertension		394 (4.98%)	132 (3.59%)	262 (6.19%)	<0.001	2 (0.16%)	0 (0.0%)	2 (0.31%)	0.156
	moderate (PA systolic 31–55 mmHg)	346 (4.37%)	113 (3.07%)	233 (5.51%)	<0.001	2 (0.16%)	0 (0.00%)	2 (0.31%)	0.156
	severe (PA systolic >55 mmHg)	48 (0.61%)	19 (0.52%)	29 (0.69%)	0.335	0 (0.0%)	0 (0.00%)	0 (0.00%)	N/A
renal impairment		2,404 (30.38%)	1,248 (33.90%)	1,156 (27.32%)	<0.001	228 (17.92%)	110 (17.29%)	118 (18.55%)	0.559
	moderate (CC >50 & <85)	1,860 (23.51%)	964 (26.19%)	896 (21.17%)	<0.001	203 (15.96%)	96 (15.09%)	107 (16.82%)	0.399
	severe (CC <50)	503 (6.36%)	263 (7.14%)	240 (5.67%)	0.007	21 (1.65%)	13 (2.04%)	8 (1.26%)	0.268
	dialysis (regardless of CC)	43 (0.54%)	21 (0.57%)	22 (0.52%)	0.760	4 (0.31%)	1 (0.16%)	3 (0.47%)	0.316
peripheral artery disease		1,519 (19.20%)	596 (16.19%)	923 (21.81%)	<0.001	142 (11.16%)	66 (10.38%)	76 (11.95%)	0.373
cerebrovascular disease		753 (9.52%)	349 (9.48%)	404 (9.55%)	0.921	77 (6.05%)	31 (4.87%)	46 (7.23%)	0.077
	history of stroke	304 (3.84%)	145 (3.94%)	159 (3.76%)	0.674	40 (3.14%)	21 (3.30%)	19 (2.99%)	0.748
	history of TIA	305 (3.85%)	173 (4.70%)	132 (3.12%)	<0.001	16 (1.26%)	8 (1.26%)	8 (1.26%)	0.999
	carotid intervention	113 (1.43%)	54 (1.47%)	59 (1.39%)	0.785	6 (0.47%)	4 (0.63%)	2 (0.31%)	0.413
chronic lung disease		694 (8.77%)	279 (7.58%)	415 (9.81%)	<0.001	49 (3.85%)	30 (4.72%)	19 (2.99%)	0.109
	asthma	295 (3.73%)	144 (3.91%)	151 (3.57%)	0.421	7 (0.55%)	5 (0.79%)	2 (0.31%)	0.255
LVEF (%) (median [IQR])*		50 [40–55]	50 [40–55]	50 [40–55]	0.031	50 [43–59]	50 [45–60]	50 [42–59]	0.456
<20%		120 (1.75%)	65 (1.94%)	55 (1.57%)	0.254	4 (0.31%)	2 (0.31%)	2 (0.31%)	0.999
21–30%		627 (9.15%)	306 (9.12%)	321 (9.19%)	0.918	8 (0.63%)	4 (0.63%)	4 (0.63%)	0.999
31–50%		3,578 (52.24%)	1,765 (52.59%)	1,813 (51.90%)	0.568	620 (48.74%)	310 (48.74%)	310 (48.74%)	0.999
>50%		2,524 (36.85%)	1,220 (36.35%)	1,304 (37.33%)	0.401	640 (50.31%)	320 (50.31%)	320 (50.31%)	0.999
CAD*									
	1 VD	692 (9.13%)	225 (6.30%)	467 (11.66%)	<0.001	103 (8.10%)	46 (7.23%)	57 (8.96%)	0.259
	2 VD	2,383 (31.45%)	1,000 (27.99%)	1,383 (34.54%)	<0.001	426 (33.49%)	203 (31.92%)	223 (35.06%)	0.235
	3 VD	4,502 (59.42%)	2,348 (65.72%)	2,154 (53.80%)	<0.001	743 (58.41%)	387 (60.85%)	356 (55.97%)	0.078
	LM disease	2,217 (29.26%)	1,034 (28.94%)	1,183 (29.55%)	0.563	324 (26.51%)	162 (26.51%)	162 (26.51%)	0.999
previous MI		4,363 (55.14%)	1,958 (53.19%)	2,405 (56.83%)	0.001	606 (47.64%)	301 (47.33%)	305 (47.95%)	0.822
	>1	871 (11.01%)	389 (10.57%)	482 (11.39%)	0.244	72 (5.66%)	36 (5.66%)	36 (5.66%)	0.999
previous PCI		1,875 (23.70%)	860 (23.36%)	1,015 (23.98%)	0.517	290 (22.80%)	151 (23.74%)	139 (21.86%)	0.423
NYHA									
	0	1,061 (13.41%)	528 (14.34%)	533 (12.59%)	0.023	273 (21.46%)	136 (21.38%)	137 (21.82%)	0.822
	I	1,655 (20.91%)	789 (21.43%)	866 (20.46%)	0.289	284 (22.33%)	142 (22.33%)	142 (22.33%)	0.999
	II	3,690 (46.63%)	1,690 (45.91%)	2,000 (47.26%)	0.231	592 (46.54%)	295 (46.38%)	297 (46.69%)	0.994
	III	1,238 (15.65%)	534 (14.51%)	704 (16.64%)	0.009	116 (9.12%)	60 (9.46%)	56 (8.80%)	0.697
	IV	269 (3.40%)	140 (3.80%)	129 (3.05%)	0.065	7 (0.55%)	3 (0.47%)	4 (0.63%)	0.706
CCS									
	0	112 (1.42%)	61 (1.66%)	51 (1.21%)	0.090	526 (41.35%)	263 (41.35%)	263 (41.35%)	0.999
	1	593 (7.49%)	362 (9.83%)	231 (5.46%)	0.000	104 (8.18%)	52 (8.18%)	52 (8.18%)	0.999
	2	2,837 (35.85%)	1,326 (36.02%)	1,511 (35.70%)	0.768	284 (22.33%)	142 (22.33%)	142 (22.33%)	0.999
	3	3,218 (40.67%)	1,397 (37.95%)	1,821 (43.03%)	<0.001	286 (22.48%)	143 (22.48%)	143 (22.48%)	0.999
	4	903 (11.41%)	419 (11.38%)	484 (11.44%)	0.940	72 (5.66%)	36 (5.66%)	36 (5.66%)	0.999
	ACS	250 (3.16%)	116 (3.15%)	134 (3.17%)	0.970	10 (0.79%)	5 (0.79%)	5 (0.79%)	0.999

*missing data

CABG, coronary artery bypass grafting; PS, propensity score; IQR, interquartile range; BMI, body mass index; PA, pulmonary artery; CC, creatinine clearance; TIA, transient ischemic attack; LVEF, left ventricle ejection fraction; CAD, coronary artery disease; VD, vessel disease; MI, myocardial infarction; PCI, percutaneous coronary intervention; NYHA, New York Heart Association; CCS, Canadian Cardiovascular Society.

Regarding clinical characteristics at time of procedure, significantly more cases of cardiogenic shock (p = 0.022), critical preoperative state (p<0.001) and insertion of IABP preoperatively (p = 0.003) were included in On-Pump CABG subset of patients. Majority of included patients were of elective- status (4,842 [61.2%]) followed by urgent- (2,858 [36.1%]) and emergency- (190 [2.4%]) with similar distribution across On-Pump and Off-Pump groups. The details on operative data is further available in Table 2.

**Table 2:** Operative characteristics before and after PS-matching.

Variable		All patients				PS-matched patients			
		Total (7,913)	On-Pump CABG (3,681)	Off-Pump CABG (4,232)	P_value_	Total (1,272)	On-Pump CABG (636)	Off-Pump CABG (636)	P_value_
***Procedural characteristics***									
	Redo surgery	84 (1.06%)	37 (1.01%)	47 (1.11%)	0.648	0 (0.00%)	0 (0.00%)	0 (0.00%)	0.999
	Cardiogenic chock	137 (1.73%)	77 (2.09%)	60 (1.42%)	0.022	3 (0.2350	1 (0.16%)	2 (0.31%)	0.563
	Critical preoperative state	198 (2.50%)	129 (3.50%)	69 (1.63%)	<0.001	0 (0.00%)	0 (0.00%)	0 (0.00%)	0.999
	IABP	148 (1.87%)	87 (2.36%)	61 (1.44%)	0.003	28 (2.20%)	16 (2.52%)	12 (1.89%)	0.444
	iv. inotropes	226 (2.86%)	117 (3.18%)	109 (2.58%)	0.109	9 (0.71%)	3 (0.47%)	6 (0.94%)	0.315
	iv. nitrates	1,264 (15.97%)	624 (16.95%)	640 (15.12%)	0.027	148 (11.64%)	72 (11.32%)	76 (11.95%)	0.726
***Urgency***									
	Elective	4,842 (61.19%)	2,241 (60.88%)	2,601 (61.46%)	0.597	918 (72.17%)	474 (74.53%)	444 (69.81%)	0.060
	Urgent	2,858 (36.12%)	1,320 (35.86%)	1,538 (36.34%)	0.656	348 (27.36%)	159 (25.00%)	189 (29.72%)	0.059
	Emergency	190 (2.40%)	101 (2.74%)	89 (2.10%)	0.064	1 (0.08%)	0 (0.00%)	1 (0.16%)	0.238
	Salvage	27 (0.34%)	19 (0.52%)	8 (0.19%)	0.014	1 (0.08%)	0 (0.00%)	1 (0.16%)	0.238
***Surgery***									
	CPB [min]*	-	84.1±42.6	NA	NA	-	79.3±32.1	NA	NA
	X-clamp [min]*	-	46.2±46.3	NA	NA	-	48.4±48.9	NA	NA
	Aortic no-touch*	1,459 (18.44%)	NA	1,459 (34.48%)	NA	0 (0.00%)	NA	0 (0.00%)	NA
	Conversion	83 (1.05%)	2 (0.05%)	81 (1.91%)	<0.001	0 (0.00%)	0 (0.00%)	0 (0.00%)	0.999
	Concomitant ablation	346 (4.37%)	209 (5.68%)	137 (3.24%)	<0.001	67 (5.27%)	35 (5.50%)	32 (5.03%)	0.706
	Concomitant LAAO	70 (0.88%)	56 (1.52%)	14 (0.33%)	<0.001	0 (0.00%)	0 (0.00%)	0 (0.00%)	0.999
	Concomitant VSD repair	19 (0.24%)	19 (0.52%)	NA	NA			NA	NA
	Concomitant ventricular aneurysm repair	45 (0.57%)	25 (0.68%)	20 (0.47%)	0.224	0 (0.00%)	0 (0.00%)	0 (0.00%)	0.999
Grafts and anastomoses*									
	LIMA	6,271 (79.25%)	2,835 (77.02%)	3,436 (81.19%)	<0.001	1,052 (82.70%)	529 (83.18%)	523 (82.26%)	0.656
	RIMA	245 (3.10%)	86 (2.34%)	159 (3.76%)	<0.001	6 (0.47%)	3 (0.47%)	3 (0.47%)	0.999
	BIMA	223 (2.82%)	81 (2.20%)	142 (3.36%)	0.002	6 (0.47%)	3 (0.47%)	3 (0.47%)	0.999
	Pedicled IMA	3,984 (61.14%)	1,876 (50.96%)	2,108 (49.81%)	0.306	1,268 (99.69%)	633 (99.53%)	635 (99.84%)	0.316
	Skeletonized IMA	2,308 (35.42%)	961 (26.11%)	1,347 (31.83%)	<0.001	364 (28.62%)	188 (29.56%)	176 (27.67%)	0.456
	Radial artery	180 (2.27%)	72 (1.96%)	108 (2.55%)	0.076	3 (0.2350	1 (0.16%)	2 (0.31%)	0.563
	Arterial anastomoses	7,940 (38.54%)	3,670 (35.19%)	4,270 (41.99%)	<0.001	444 (34.91%)	221 (34.75%)	223 (35.06%)	0.906
	Venous anastomoses	11,004 (53.42%)	6,076 (58.26%)	4,928 (48.46%)	<0.001	615 (49.35%)	310 (48.74%)	305 (47.95%)	0.779
	Sequential anastomoses	1,656 (8.04%)	684 (6.56%)	972 (9.56%)	<0.001	21 (1.65%)	11 (1.73%)	10 (1.57%)	0.826
	Composite anastomoses	620 (3.01%)	257 (2.46%)	363 (3.57%)	<0.001	2 (0.16%)	1 (0.16%)	1 (0.16%)	0.999
	Total arterial revascularization	1,555 (19.65%)	344 (9.35%)	1,211 (28.62%)	<0.001	207 (16.27%)	103 (16.19%)	104 (16.35%)	0.939

*missing data

CABG, coronary artery bypass grafting; PS, propensity score; IABP, intra-aortic balloon pump; iv, intravenous; OPCAB, Off-Pump Coronary Artery Bypass; CPB, cardiopulmonary bypass; LAAO, left atrial appendage occlusion; VSD, ventricular septal defect; LIMA/RIMA/BIMA, Left/Right/Bilateral Internal Mammary Artery; NA, not available.

### Operative and long-term data

Average CPB time was estimated at 84.1±42.6 minutes, while X-clamp was 46.2±46.3 minutes. Concomitant AF ablation was performed significantly more often in On-Pump CABG group as compared to Off-Pump CABG (209 [5.7%] vs 137 [3.2%]; P<0.001). Left internal mammary artery (LIMA) grafts were used in 79.3% of cases and more frequently in Off-Pump CABG (81.2% vs 77.0%, p<0.001); pedicled IMA was harvested almost twice as often as skeletonized IMA (61.1% vs 35.4%). Complete revascularization was possible in 67.5% of patients and was significantly higher, by 10%, in patients undergoing On-Pump CABG (73.3% vs 62.6%; P<0.001). While arterial anastomoses accounted for 38.5% of all distal anastomoses, total arterial revascularization was achieved in 19.6% of overall population with over three-fold higher rates in Off-Pump CABG (p<0.001). Aortic no-touch technique was used in 34.5% of Off-Pump cases. Conversions from Off-Pump to On-Pump followed in 81 patients accounting for 1.9%.

Median (IQR) HLoS was 10 (8–14) days and ICU stay was 1.17 (0.91–1.50). The HLoS was significantly longer in On-Pump CABG as compared to Off-Pump (std. mean diff. [95%CIs] 0.166 [0.121, 0.210] day or 3.98 [2.90–5.04] hours, p<0.001) and so was length of ICU stay (std. mean diff. [95%CIs] 0.212 [0.168, 0.257] day or 5.09 [4.03–6.17] hours, p<0.001).

**Fig 2:**
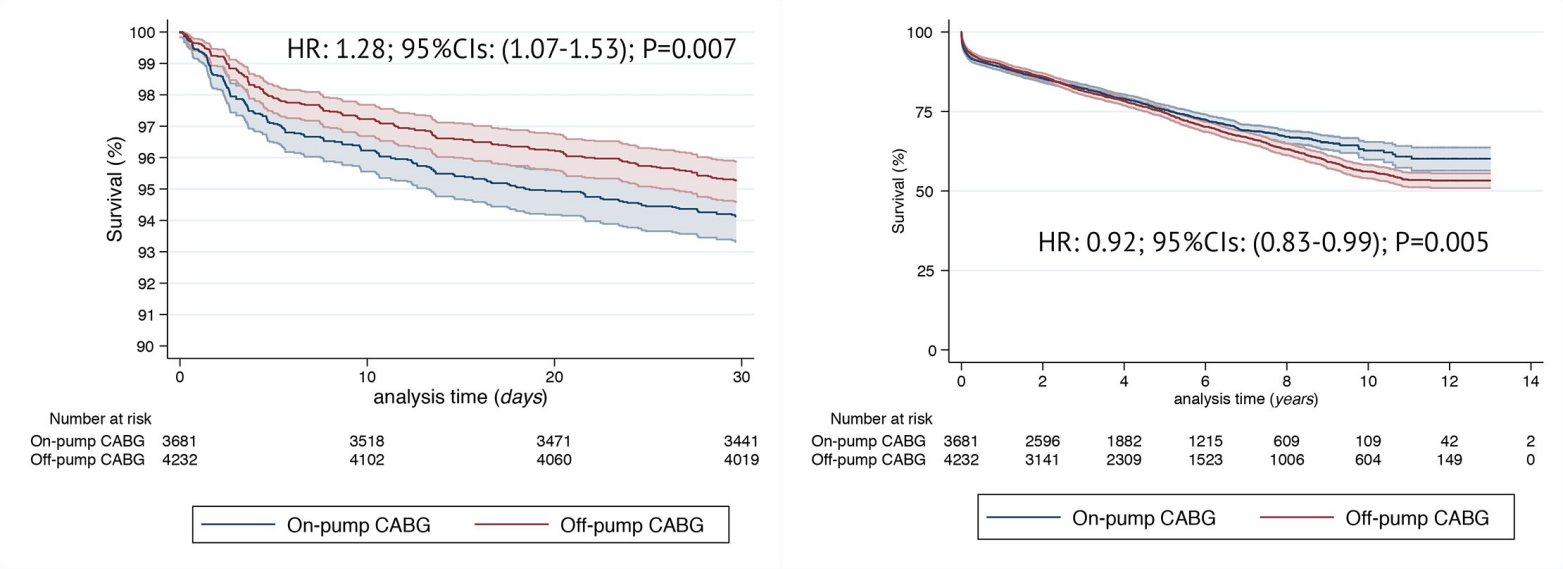
Unadjusted Kaplan-Meier survival curves between the two groups: On-Pump and Off-Pump CABG for the analysis of 30-day (A) and late (B) survival. Hazard Ratios and respective 95% Confidence Intervals in the On-Pump as compared to Off-Pump CABG.

In unadjusted analysis, On-Pump CABG was associated with increased rates of postoperative complications, among them cardiac tamponade and/or rethoracotomy for bleeding, periprocedural MI, respiratory and multiorgan failure, gastrointestinal complications and acute kidney injury. Early postoperative mortality was significantly higher in On-Pump CABG: HR 1.74; 95%CI (1.09–2.78); p = 0.019. At 30-days On-Pump CABG was associated with significant nearly 30% increased mortality risk: HR 1.28; 95%CI (1.07–1.53); p = 0.007. Fig 2A. Within investigated follow-up unadjusted HR for long term survival, however, favored On-Pump CABG: HR 0.92; 95%CI (0.83–0.99); p = 0.005 (Fig 2B and S1 File). List of remaining in-hospital outcomes is available as Table 3.

**Table 3:** In-hospital outcomes before and after PS-matching.

	All patients				PS-matched patients			
	On-Pump CABG (3,681)	Off-Pump CABG (4,232)	Hazard Ratio (95%CIs)	P_value_	On-Pump CABG (636)	Off-Pump CABG (636)	Hazard Ratio (95%CIs)	P_value_
Early postoperative mortality	44 (1.20%)	29 (0.69%)	1.74 (1.09–2.78)	0.019	8 (1.25%)	1 (0.16%)	8.00 (1.01–63.78)	0.049
30-day mortality	238 (6.47%)	214 (5.06%)	1.28 (1.07–1.53)	0.007	18 (2.83%)	5 (0.79%)	3.58 (1.34–9.61)	0.001
Cardiac tamponade and/or rethoracotomy	226 (6.14%)	177 (4.18%)	1.47 (1.21–1.78)	<0.001	44 (6.92%)	29 (4.56%)	1.51 (0.96–2.39)	0.073
Periprocedural MI	53 (1.44%)	37 (0.87%)	1.65 (1.08–2.50)	0.019	2 (0.31%)	2 (0.31%)	1.00 (0.14–7.07)	0.999
Respiratory failure	255 (6.93%)	243 (5.74%)	1.21 (1.02–1.43)	0.031	31 (4.87%)	25 (3.93%)	1.24 (0.74–2.07)	0.413
Prolonged ICU stay	50 (1.36%)	64 (1.51%)	0.90 (0.62–1.30)	0.567	3 (0.47%)	9 (1.42%)	0.33 (0.91–1.22)	0.098
Neurologic complications	93 (2.53%)	90 (2.13%)	1.19 (0.89–1.58)	0.238	2 (0.31%)	2 (0.31%)	1.00 (0.14–7.07)	0.999
Multiorgan failure	99 (2.69%)	79 (1.87%)	1.44 (1.08–1.93)	0.014	6 (0.94%)	8 (1.26%)	0.75 (0.26–2.15)	0.592
Gastrointestinal complications	69 (1.87%)	50 (1.18%)	1.59 (1.11–2.28)	0.012	4 (0.63%)	7 (1.10%)	0.57 (0.16–1.54)	0.370
Acute kidney failure and/or dialysis	146 (3.97%)	95 (2.24%)	1.77 (1.37–2.28)	<0.001	6 (0.94%)	8 (1.26%)	0.75 (0.26–2.15)	0.592
Superficial sternal wound infection	65 (1.77%)	78 (1.84%)	0.96 (0.69–1.33)	0.797	7 (1.10%)	9 (1.42%)	0.77 (0.29–2.07)	0.616
Deep sternal wound infection	47 (1.28%)	48 (1.13%)	1.13 (0.75–1.68)	0.561	8 (1.26%)	4 (0.63%)	2.00 (0.61–6.61)	0.256
Mediastinitis	30 (0.81%)	24 (0.57%)	1.44 (0.84–2.45)	0.184	3 (0.47%)	2 (0.31%)	1.50 (0.25–8.94)	0.656
PPI	10 (0.27%)	9 (0.21%)	1.28 (0.52–3.14)	0.594	0 (0.00%)	0 (0.00%)	1.00 (0.02–50.32)	0.999
ECMO	11 (0.30%)	2 (0.05%)	6.32 (1.4–28.51)	0.016	1 (0.16%)	0 (0.00%)	3.00 (0.12–73.51)	0.501
IABP	193 (5.24%)	156 (3.69%)	1.42 (1.16–1.75)	<0.001	16 (2.52%)	12 (1.89%)	1.33 (0.64–2.80)	0.446

CABG, coronary artery bypass grafting; PS, propensity score; CIs, confidence intervals; MI, myocardial infarction; ICU, intensive care unit; PPI, permanent pacemekar implantation; ECMO, extracorporeal membrane oxygenation; IABP, intra-aortic balloon pump.

### Propensity score analysis

**Fig 3:**
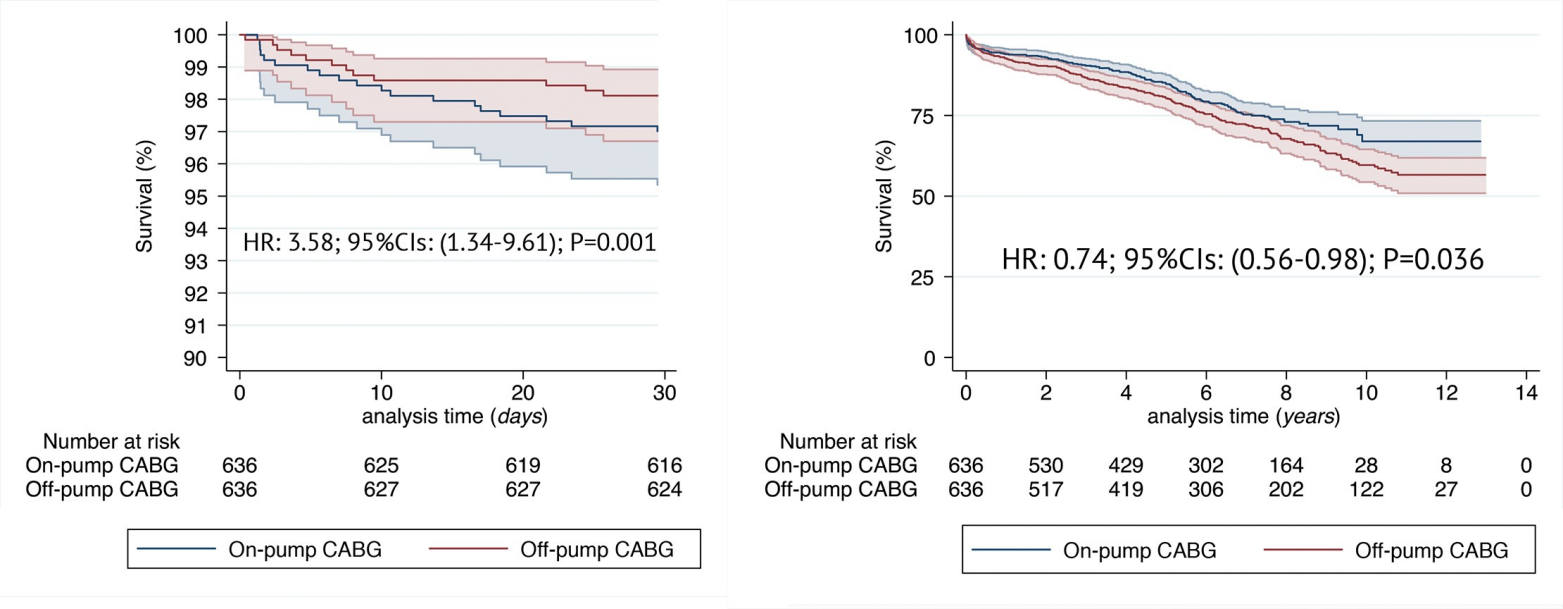
Propensity matched Kaplan-Meier survival curves between the two groups: On-Pump and Off-Pump CABG for the analysis of 30-day (A) and late (B) survival. Hazard Ratios and respective 95% Confidence Intervals in the On-Pump as compared to Off-Pump CABG.

One-to-one propensity score–matched analysis resulted in 636 pairs with similar baseline characteristics and operative covariates (Tables 1 and 2). List of variables contributing to PS along with respective propensity scores is available as S1 Table. Detailed analysis of standardized mean differences (SMDs) before and after propensity score matching comparing covariate values for patients undergoing On-Pump vs Off-Pump CABG (S1 Fig) revealed that SMDs for the measured covariates were mostly <0.1, suggesting covariate balance across groups. PS-matched 30-day mortality was increased with On-Pump CABG and estimated to HR: 3.58; (95%CIs: 1.34–9.61); p = 0.001. Fig 3A. Late mortality, on the other hand, was reduced in patients undergoing On-Pump CABG (HR: 0.74; [95%CIs: 0.56–0.98]; p = 0.036). Fig 3B. Hospital outcomes adjusted for PS are reported in Table 3. Among them a trend towards more frequent incidence of cardiac tamponade and/or rethoracotomy was observed in On-Pump CABG (HR: 1.51; [95%CIs: 0.96–2.39]; p = 0.073).

### Sensitivity and subgroup analyses

**Fig 4:**
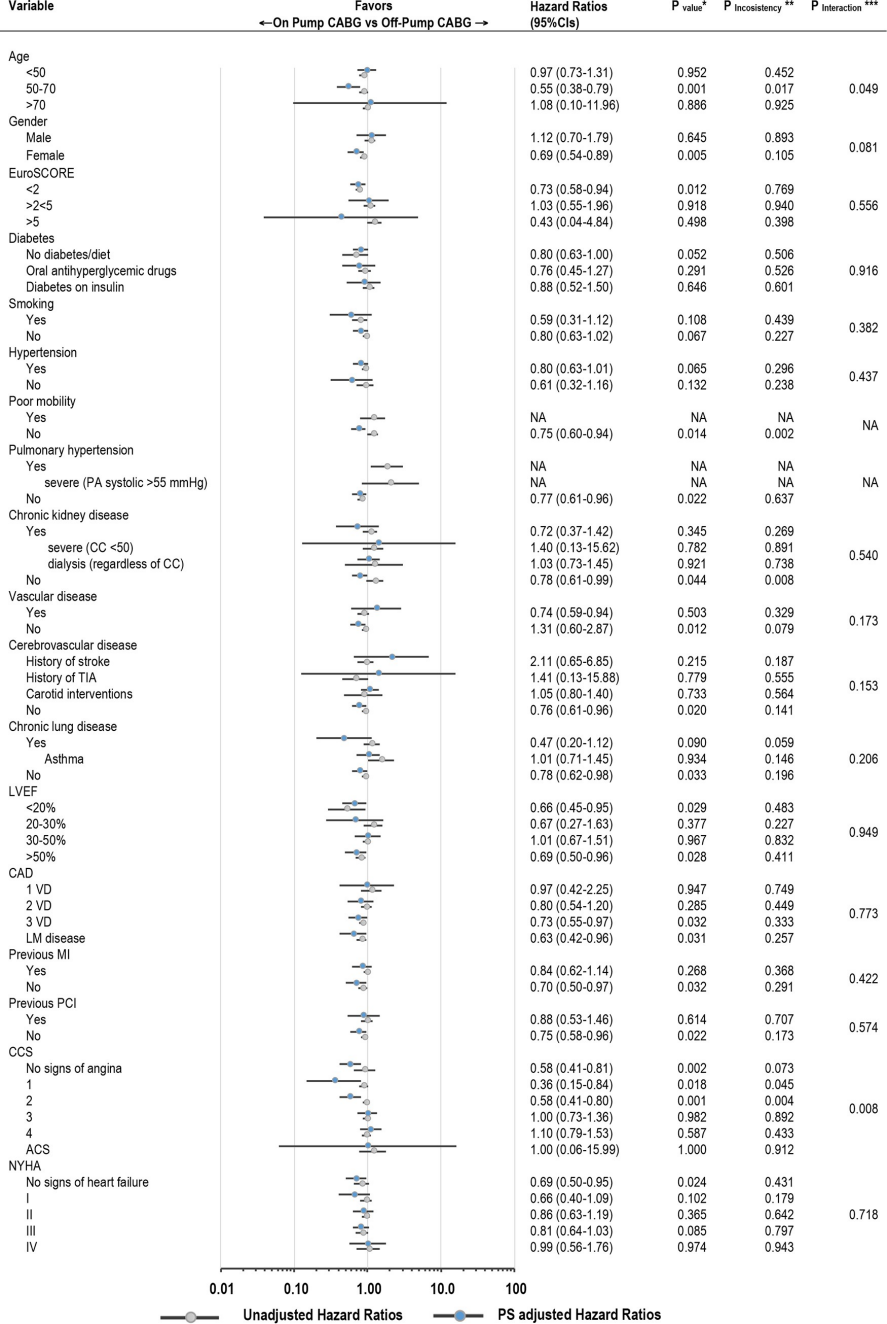
Hazard ratios and 95% confidence intervals for death from any cause in the On-Pump as compared to Off-Pump CABG according to selected preoperative baseline characteristics. CABG, coronary artery bypass grafting; PA, pulmonary artery; CC, creatinine clearance; TIA, transient ischemic attack; LVEF, left ventricle ejection fraction; CAD, coronary artery disease; VD, vessel disease; LM, left main; MI, myocardial infarction; PCI, percutaneous coronary intervention; CCS, Canadian Cardiovascular Society; ACS, acute coronary syndrome; NYHA, New York Heart Association; PS, propensity score; NA, not available. * P value for the treatment effect. ** P value for the interaction between pre- and post PS-matching estimates. *** P value for the interaction between subgroup components (after PS-matching).

**Fig 5:**
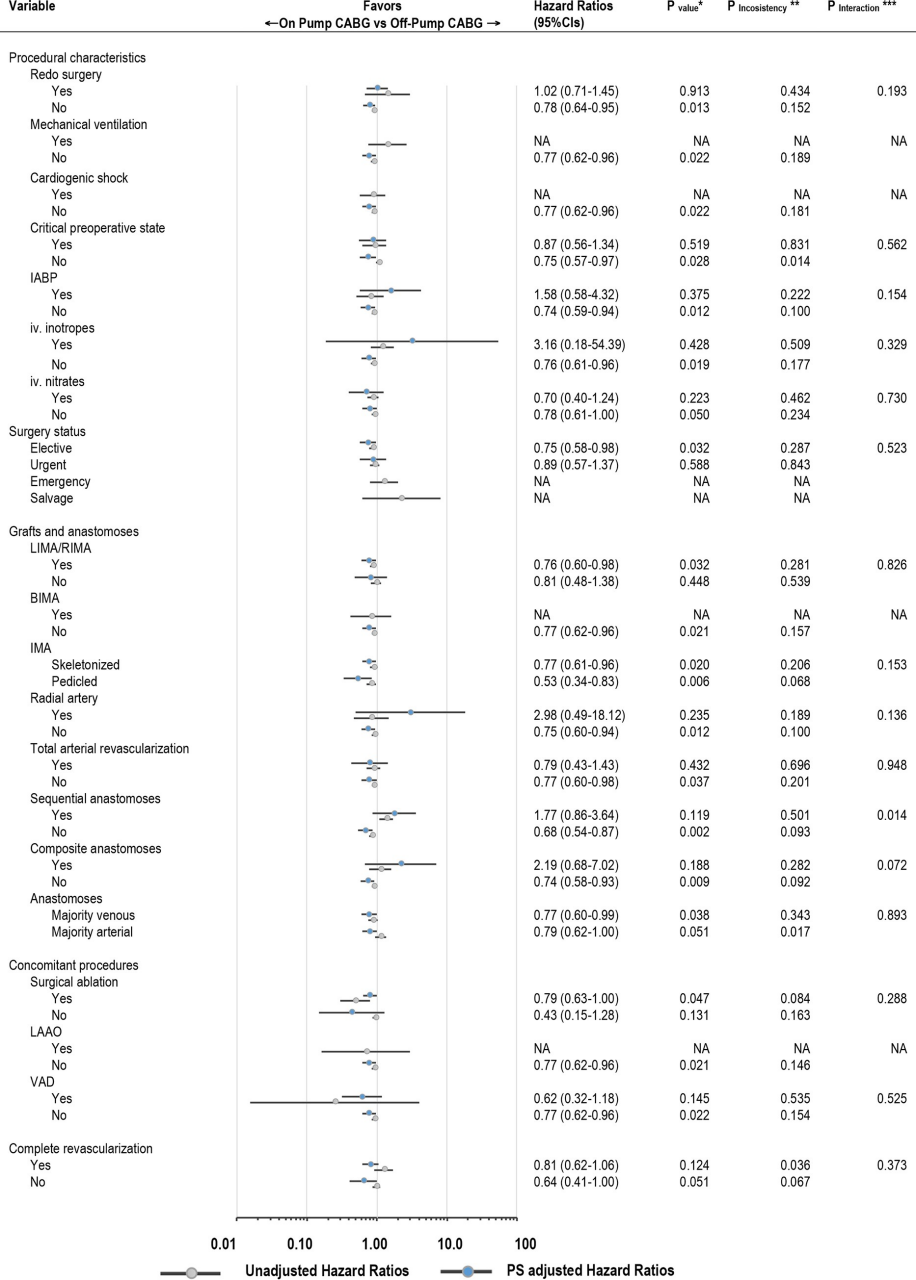
Hazard ratios and 95% confidence intervals for death from any cause in the On-Pump as compared to Off-Pump CABG according to selected procedural characteristics. IABP, intra-aortic balloon pump; iv, intravenous; LIMA/RIMA/BIMA, left/right/bilateral internal mammary artery. LAAO, left atrial appendage occlusion; VAD, ventricle assist device. Remaining abbreviations as in Fig 4.

Number of subgroup analyses were performed for comparison On-Pump vs Off-Pump before and after PS-matching (Figs 4 and 5) with respect to the primary endpoint late survival. In these analyses only two significant interactions with baseline (Fig 4) or procedural (Fig 5) variables were demonstrated; indeed, the benefit of On-Pump CABG was more gradually less pronounced with growing CCS scale (P = 0.008), (Fig 4); operatively, there was higher extent of survival benefit in Off-Pump CABG when both sequential and/or composite anastomoses were performed (P = 0.014 and P = 0.072 respectively); On-Pump CABG was beneficial in case these were not used (P = 0.009 and P = 0.051), (Fig 5). After PS-matching, the direction of benefit with On-Pump CABG was maintained across subgroups of patients as compared to unadjusted estimates, yet was particularly present in patients with lower baseline surgical risk such as those with EuroSCORE <2; preserved ejection fraction and without comorbidities. The detailed analysis with reporting for both univariable and multivariable Cox proportional hazards model is appended as S2 Table.

## Discussion

With all limitations and selection bias inherent to registry analyses, that were however taken account for by matching for propensity scores, the current report from one of Europe’s largest registries on heart surgery procedures shows robust evidence to support as follows: in patients with underlying AF and undergoing CABG 1) off-pump CABG was associated with improved short-term survival; 2) over the study course there was a shift in survival favoring on-pump CABG in the long-term; 3) performing sequential/composite anastomoses yielded survival benefits during off-pump CABG.

Although the presence of AF in patients undergoing CABG is much less than their MV surgery counterparts, still approximately 6% of patients presenting for coronary surgery have preoperative AF [1,3] regardless of the AF origin (eg. valvular vs non-valvular). Preoperative AF was found to be associated with a higher adjusted 30-day mortality and morbidity including stroke, renal failure, prolonged ventilation, reoperation, and deep sternal wound complications; patients with preoperative AF also experience a higher adjusted long-term risk of all-cause mortality and cumulative risk of stroke and systemic embolism compared to those without AF [21,22]. Said that, AF patients undergoing CABG, regardless of the initial EuroSCORE may be considered high risk.

Concept of risk gradient in CABG, and in particular in comparison On-Pump CABG vs Off-Pump has been well defined [8]. Yet controversies remain as while there have been studies showing the potential benefits of OPCAB in this sub-group of patients, there is still a lot to be learned from the patient selection and application of this technique. Previous observational studies suggested that, by avoiding the negative effects of cardiopulmonary bypass, off-pump CABG may substantially reduce mortality and morbidity rates when compared with conventional on-pump CABG [6,23,24]. These benefits, however, have never been confirmed in a single randomized controlled trial (RCT). Veterans Affairs Randomized On/Off Bypass (ROOBY) trial [25] demonstrated no significant difference between off-pump and on-pump CABG in the incidence of the 30-day composite endpoint of death, reoperation, new mechanical support, cardiac arrest, coma, stroke, or renal failure (7.0% and 5.6%, respectively; P = 0.19). Similarly, the largest trial to date, the CABG Off or On Pump Revascularization Study (CORONARY) [26] which included more than 4700 patients randomized to OPCAB and conventional CABG, showed no difference between these 2 approaches with regard to 30-day rate of mortality, myocardial infarction (MI), stroke, or renal failure requiring dialysis. Indeed, the only RCT designed to address “elevated” risk patients exclusively was the German Off-Pump Coronary Artery Bypass Grafting in Elderly Patients (GOPCABE) trial which also found that in elderly patients ≥75 years, five-year survival rates as well as the combined outcome of death, MI and repeat revascularization were similar after on-pump and off-pump CABG [27]. It is the GOPCABE investigators who recently issued a post-hoc sub-analysis of off-pump vs on-pump trial focusing on patients with preoperative AF [13]. With similar prevalence of AF across subgroups (10% on-pump, 10% off-pump), AF patients, as expected, had worse preoperative conditions, which, in turn, had a negative impact on outcome; combined end-point of death, MI, stroke, dialysis and revascularization occurred more often (13 vs 8%, P = 0.008) and 30-day mortality was significantly higher (6 vs 2%, P = 0.003) in AF patients. However, the operative technique used for CABG did not affect these outcome parameters. Neutrality of these results must be viewed in light of low mortality and other complications’ rates in patients undergoing CABG nowadays and, in particular, in patients selected for enrollment in a RCT. On the other hand, current analysis is placed in all-comers scenario and allowed for inclusion of almost 8,000 AF patients undergoing CABG which is more than any RCT (233 pts. In GOPCABE) or RCTs combined ever had.

First and most important finding of the current report is that as compared to Off-Pump, On-Pump CABG was associated with higher early (24 hours) and 30-day all-cause mortality after PS matching (HR [95%CIs]: 8.00 [1.01–63.78] P = 0.049; and 3.58 [1.34–9.61] P = 0.001 respectively). The finding is in line with another report from Attaran [12] that while not powered for hard clinical endpoints, demonstrated that patients undergoing on-pump CABG, presence of underlying AF was associated with poorer postoperative outcomes; inotropic support rates as well as need for IABP support remained significantly higher in AF, even after adjusting for preoperative characteristics. Authors suggested more apparent and negative effect of CPB in patients with AF at short-term; even despite maintaining acceptable perfusion pressures during CPB. Generalized hypoperfusion [28] together with deleterious effects of CPB itself further increases rates of postoperative complications and early mortality. Before adjusting for PS in the current analysis, patients undergoing On-Pump CABG experienced more cardiac tamponades/reoperations for bleeding, periprocedural MIs, respiratory and multiorgan failures, acute kidney injuries and ECMO/IABP support; yet, these differences were no longer prominent after matching.

Secondly, late mortality, on the other hand, was reduced in patients undergoing On-Pump CABG (HR: 0.74; [95%CIs: 0.56–0.98]; p = 0.036). In the present analysis we were able to show for the first time a shift in the survival which favored Off-Pump in the short term and then On-Pump in the remote observations leading to conclusions that factors other than baseline characteristics play a role in forging the late postoperative course. Completeness of revascularization has long been claimed to influence remote survival after CABG [10,11,29] and present report is no exception. A completeness of revascularization index (CRI) was calculated based on the difference between the number of coronary grafts and the number of diseased coronary artery systems as reported in the KROK database; CR was more frequently achieved in the On-Pump CABG group (73.3% vs 62.6%; P<0.001); again, this reflects findings of recent studies confirming OPCAB being associated with less grafts per patient and less complete revascularization [29–31]. Since the above were found to be linked to increased incidence of recurrent angina, need for repeat revascularization procedures (both PCI and re-CABG), and more frequent rehospitalization for cardiac-related issues, particular over the course of mid-term follow-up [9], achieving CR and in particular in high-risk patient is of paramount importance. Experience factor, not accounted for in the current analysis, is not to be missed however, since when multiple arterial grafts are used and a complete revascularization is performed in centres experienced in OPCAB or performing OPCABs only, reported outcomes are equivalent to those of ONCAB procedures [32–34].

Third and unexpected finding of the current analysis is the significant interaction between survival estimates in On-Pump vs Off-Pump CABG when composite and sequential anastomoses were performed. We were able to demonstrate that whenever these were not used during On-Pump translated into improved survival; on the other hand, a non-significant yet ‘not to be missed’ trend for improved survival with Off-Pump CABG was seen in cases composite and sequential anastomoses were performed; it is hard to discuss on potential explanations for this phenomenon in the absence of angiographic follow-up; yet it may be assumed that given higher global number of venous anastomoses in the On-Pump (58.3% vs 48.5%) and arterial- in the Off-Pump-CABG (41.2% vs 35.2%), the quality of sequential and/or composite venous anastomosis is far inferior to one arterial- over time, leading to differences in survival estimates between the two groups.

Interpreting the above, caution must be used however with regard to low reported rates of surgical ablation since both early and late patency of the grafts seems to be greatly influenced by postoperative rhythm; in the studies assessing blood flow through the grafts, AF caused significant deterioration in hemodynamics: heart rate and central venous pressure increased, and mean arterial pressure and cardiac index decreased (P = 0.003). In LIMA grafts, the flow decreased significantly in AF (P<0.001) as measured using transit-time flowmetry [35,36]. By avoiding postoperative AF, the vulnerable grafts are protected against diastolic impairment and low cardiac output syndrome that is prominent in case of arrhythmias [37] and are less likely to fail. Surgical ablation concomitant to CABG surgery was performed in 4.4% in the overall population (5.7% On-Pump vs 3.2% Off-Pump CABG) and is much lower prevalence than observed in Society of Thoracic Surgery reports (17%-30%) [1,3] which we find disturbing. Yet, ablation procedures concomitant to CABG are not reimbursed neither in Poland nor in majority of European countries [38]; therefore, surgical ablation at time of isolated CABG is performed at physicians’ discretion or driven by industry funded research programs. Latest report from the KROK registry has found that surgical ablation performed for AF at time of isolated CABG was associated with significantly improved remote survival [39]; therefore, addition of arrhythmias correction surgery could have further improved the survival rates equally (P _interaction_ = 0.228) in both On-Pump and Off-Pump CABG in the current analysis.

Current study purpose was not to end a “On-pump vs Off-pump never ending debate”, rather it adds fuel to it pointing to certain limitations and advantages of one approach over the other in the setting of AF. From the largest CABG and AF multicenter registry to-date, it seems reasonable to individualize the treatment and tailor the CABG surgery to the AF patient, having in mind that Off-Pump CABG confers short term survival benefits and should strongly be considered in high risk patient. On the other hand, patients in the lower risk groups, with otherwise longer life expectancy will survive to benefit from higher rates of complete revascularization offered by On-Pump CABG.

### Limitations

Limitations of the KROK registry have been described before [14]. Completeness of revascularization rates are lower than what is observed in STS reports [1–3]. Contributing to lower rates of CR in the Off-Pump CABG group is the fact that minimally invasive direct coronary artery bypass (MIDCABs) were not excluded from the analysis. Hybrid procedures (MIDCAB: LIMA-LAD followed by PCI to remaining lesions) is widely performed and in particular recently. Index of complete revascularization, on the other hand, was calculated based on the difference between the number of coronary grafts and not target lesions revascularization. Secondly, we could not account for left atrial appendage (LAA) closure rates; in the current report these are certainly underscored; further, ablation- durations, techniques and -immediate success rates since these were not obligatory to complete during registry conception as well and these data are incomplete. LIMA grafts were used in only 80% of patients, yet it must be noted, the registry covers an almost 15-year time span and included both elective and emergency CABG procedures together with ACS, dialysis and redo surgeries. Finally, since the registry is anonymous, we could not adjust for centers’ and surgeons’ volume and experience with one technique or another.

## Conclusions

Off-Pump CABG offered 30-day survival benefit to patients undergoing CABG surgery and presenting with underlying AF. On-Pump CABG was associated with significantly improved survival at long term.

## Supporting information

S1 Fig Standardized differences before and after propensity score matching comparing covariate values for patients undergoing Off-Pump and On-Pump CABG. 3VD. three vessel disease; IABP. intra-aortic balloon pump; TIA. transient ischemic attack; CCS. Canadian Cardiovascular Society; LVEF. left ventricle ejection fraction; CKD. chronic kidney disease; NYHA. New York Heart Association; DM. diabetes mellitus; BMI. body mass index; HT. hypertension; IMA. internal mammary artery; LM. left main; ACS. acute coronary syndrome CVD. cerebrovascular disease; PCI percutaneous coronary intervention; MI. myocardial infarction; PHT. pulmonary hypertension; PAD. peripheral artery disease; BIMA. bilateral internal mammary artery; RIMA. right internal mammary artery; TAR. total arterial revascularization.
(PDF)

S1 Table Variables contributing to propensity score-matching along with respective propensity scores. PAD. peripheral artery disease; CVD. cerebrovascular disease; TIA. transient ischemic attack; CAD. coronary artery disease; LVEF. left ventricle ejection fraction; IABP. intra-aortic balloon pump; LAAO. left atrial appendage occlusion.
(PDF)

S2 Table Cox Proportional Hazard Univariable and Multivariable Model estimates before and after PS-matching. HR, hazard ratio; CIs, confidence intervals; PHT, pulmonary hypertension; CKD, chronic kidney disease; PAD, peripheral artery disease; TIA, transient ischemic attack; LVEF, left ventricle ejection fraction; NYHA, New York Heart Association; CCS, Canadian Cardiovascular Society; CAD, coronary artery disease; LM, left main; MI, myocardial infarction; PCI, percutaneous coronary intervention; IABP, intra-aortic balloon pump; IMA/RIMA/BIMA, right/bilateral internal mammary artery; TAR, total arterial revascularization; LAAO, left atrial appendage occlusion.
(PDF)

S1 File (XLSX)

## List of abbreviations

CABG: coronary artery bypass grafting
AF: atrial fibrillation
CPB: cardiopulmonary bypass
ECC: extracorporeal circulation
MV: mitral valve
STS: Society of Thoracic Surgeons
KROK: ‘Krajowy Rejestr Operacji Kardiochirurgicznych’ (Polish National Registry of Cardiac Surgery Procedures)
LVEF: left ventricle ejection fraction
CAD: coronary artery disease
MI: myocardial infarction
PCI: percutaneous coronary intervention
IABP: intra-aortic balloon pump
ICU: intensive care unit
HLoS: hospital length of stay
HR: hazard ratio
CI: confidence interval
SMD: standardized mean difference
