# Supplementary figures and images for: On-Pump vs Off-Pump coronary artery bypass surgery in atrial fibrillation. Analysis from the polish national registry of cardiac surgery procedures (KROK)
Source: PLoS One. 2020 Apr 22;15(4):e0231950. doi: 10.1371/journal.pone.0231950 (PMC7176119; doi:10.1371/journal.pone.0231950)

Unadjusted SMD

Post PS matching SMD

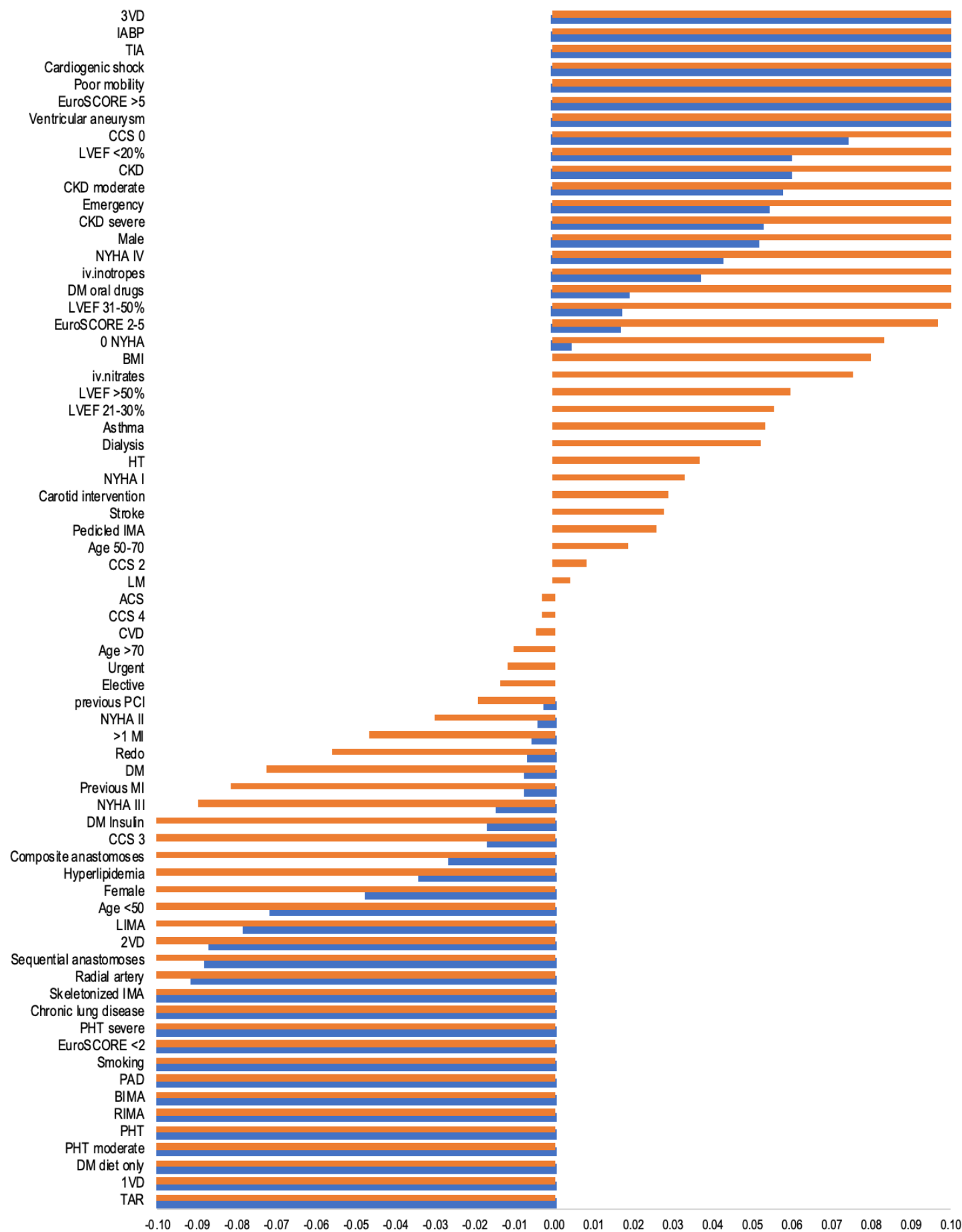

Supplement: S1 Fig — 3VD. three vessel disease; IABP. intra-aortic balloon pump; TIA. transient ischemic attack; CCS. Canadian Cardiovascular Society; LVEF. left ventricle ejection fraction; CKD. chronic kidney disease; NYHA. New York Heart Association; DM. diabetes mellitus; BMI. body mass index; HT. hypertension; IMA. internal mammary artery; LM. left main; ACS. acute coronary syndrome CVD. cerebrovascular disease; PCI percutaneous coronary intervention; MI. myocardial infarction; PHT. pulmonary hypertension; PAD. peripheral artery disease; BIMA. bilateral internal mammary artery; RIMA. right internal mammary artery; TAR. total arterial revascularization. (PDF) [file pone.0231950.s001.pdf]
