# Supplementary material for: On-Pump vs Off-Pump coronary artery bypass surgery in atrial fibrillation. Analysis from the polish national registry of cardiac surgery procedures (KROK)
Source: PLoS One. 2020 Apr 22;15(4):e0231950. doi: 10.1371/journal.pone.0231950 (PMC7176119; doi:10.1371/journal.pone.0231950)
Supplement: S1 Table — PAD. peripheral artery disease; CVD. cerebrovascular disease; TIA. transient ischemic attack; CAD. coronary artery disease; LVEF. left ventricle ejection fraction; IABP. intra-aortic balloon pump; LAAO. left atrial appendage occlusion. (PDF) [file pone.0231950.s002.pdf]

Table S1. Variables contributing to propensity score-matching along with respective propensity scores

Variable	Wald/χ^2	SE	Score	95%CI Lower	95%CI Upper	OR	95%CI Lower	95%CI Upper	P value
Gender	4.344	0.063	-0.132	-0.256	-0.008	0.768	0.599	0.984	0.037
Asthma	7.107	0.126	0.337	0.089	0.585	1.963	1.195	3.222	0.008
PAD	19.812	0.065	0.288	0.161	0.414	1.777	1.380	2.290	<<0.001
CVD	5.657	0.106	-0.252	-0.460	-0.044	0.604	0.399	0.915	0.017
TIA	5.870	0.143	0.345	0.066	0.625	1.995	1.141	3.489	0.015
Poor mobility	11.270	0.089	0.300	0.125	0.475	1.822	1.284	2.587	<0.001
CAD extent	34.320	0.109	-0.640	-0.854	-0.426	0.284	0.202	0.400	<<0.001
LVEF < 20	5.075	0.231	0.520	0.068	0.972	2.826	1.145	6.980	0.024
Mechanical ventilation	4.590	0.704	1.509	0.129	2.890	20.462	1.293	323.761	0.032
IABP	9.445	0.296	0.910	0.330	1.490	6.173	1.934	19.706	0.002
Ablation	24.666	0.150	0.747	0.452	1.041	4.452	2.469	8.025	<<0.001
LAAO	4.050	0.289	0.581	0.015	1.148	3.199	1.031	9.927	0.044
Conversion	6.764	0.267	0.696	0.171	1.220	4.020	1.409	11.471	0.009
EuroSCORE	27.094	0.006	0.031	0.019	0.042	1.031	1.019	1.043	<<0.001
