# Supplementary material for: On-Pump vs Off-Pump coronary artery bypass surgery in atrial fibrillation. Analysis from the polish national registry of cardiac surgery procedures (KROK)
Source: PLoS One. 2020 Apr 22;15(4):e0231950. doi: 10.1371/journal.pone.0231950 (PMC7176119; doi:10.1371/journal.pone.0231950)
Supplement: S2 Table — HR, hazard ratio; CIs, confidence intervals; PHT, pulmonary hypertension; CKD, chronic kidney disease; PAD, peripheral artery disease; TIA, transient ischemic attack; LVEF, left ventricle ejection fraction; NYHA, New York Heart Association; CCS, Canadian Cardiovascular Society; CAD, coronary artery disease; LM, left main; MI, myocardial infarction; PCI, percutaneous coronary intervention; IABP, intra-aortic balloon pump; IMA/RIMA/BIMA, right/bilateral internal mammary artery; TAR, total arterial revascularization; LAAO, left atrial appendage occlusion. (PDF) [file pone.0231950.s003.pdf]

Table S2. Cox Proportional Hazard Univariate and Multivariate Model estimates before and after PS-matching.

**Model**	**Univariable**				**Multivariable**			
**Estimates**	**Unadjusted Hazard Ratios**		**After PS-matching Hazard Ratios**		**Unadjusted Hazard Ratios**		**After PS-matching Hazard Ratios**	
**Variables**	**HR (95% CIs)**	**P**	**HR (95% CIs)**	**P**	**HR (95% CIs)**	**P**	**HR (95% CIs)**	**P**
Age	1.04 (1.04-1.05)	<0.001	1.05 (1.04-1.07)	<0.001	-	-	-	-
Male	0.99 (0.89-1.03)	0.771	0.99 (0.76-1.28)	0.918	-	-	-	-
Female	1.01 (0.92-1.12)	0.771	1.01 (0.78-1.32)	0.918	-	-	-	-
Euroscore	1.11 (1.11-1.11)	<0.001	1.27 (1.09-1.47)	0.002	1.15 (1.11-1.18)	<0.001	-	-
No diabetes/diet	0.78 (0.72-0.85)	<0.001	0.95 (0.13-6.75)	0.958	-	-	-	-
Oral antihyperglycemic drugs	1.00 (0.89-1.11)	0.948	1.07 (0.81-1.42)	0.622	-	-	-	-
Diabetes on insulin	1.49 (1.34-1.64)	<0.001	1.25 (0.94-1.66)	0.132	-	-	-	-
Smoking	1.01 (0.89-1.15)	0.823	1.17 (0.85-1.63)	0.336	-	-	-	-
Hypertension	0.95 (0.83-1.09)	0.496	0.79 (0.57-1.10)	0.166	-	-	-	-
Hyperlipidemia	0.96 (0.88-1.04)	0.293	0.85 (0.68-1.06)	0.145	-	-	-	-
Poor mobility	1.68 (1.37-2.07)	<0.001	0.93 (0.30-2.90)	0.896	-	-	-	-
PHT	1.23 (0.95-1.60)	0.113	-	-	-	-	-	-
PHT severe	3.26 (2.11-5.03)	<0.001	-	-	190.69 (86.80-418.93)	<0.001	-	-
CKD	2.07 (1.83-2.33)	<0.001	2.04 (1.41-2.99)	<0.001	-	-	-	-
Dialysis	3.47 (2.41-5.01)	<0.001	-	-	4.74 (2.24-10.05)	<0.001	-	-
PAD	1.61 (1.46-1.79)	<0.001	1.33 (0.96-1.05)	0.091	-	-	-	-
Cerebrovascular disease	1.39 (1.22-1.58)	<0.001	1.28 (0.85-1.19)	0.257	-	-	-	-
History of stroke	1.15 (0.93-1.41)	0.195	1.38 (0.79-1.26)	0.258	-	-	-	-
History of TIA	1.13 (0.92-1.39)	0.255	1.26 (0.40-3.94)	0.688	-	-	-	-
Carotid interventions	1.71 (1.28-2.29)	<0.001	1.49 (0.21-10.59)	0.690	-	-	-	-
Chronic lung disease	1.55 (1.36-1.77)	<0.001	1.95 (1.25-2.66)	0.003	-	-	1.77 (1.11-2.84)	0.016
Asthma	1.47 (1.20-1.81)	<0.001	1.45 (0.46-2.17)	0.531	-	-	-	-
LVEF	0.98 (0.98-0.99)	<0.001	0.97 (0.96-0.98)	<0.001	-	-	-	-
NYHA 0	0.72 (0.64-0.82)	<0.001	0.68 (0.50-0.92)	0.013	-	-	-	-
NYHA I	0.83 (0.75-0.92)	<0.001	0.79 (0.61-1.03)	0.086	-	-	-	-
NYHA II	0.90 (0.83-0.98)	0.013	1.19 (0.96-1.49)	0.118	-	-	-	-
NYHA III	1.57 (1.41-1.74)	<0.001	1.64 (1.19-2.27)	0.003	-	-	-	-
NYHA IV	3.25 (2.71-3.89)	<0.001	2.69 (1.00-7.23)	0.049	-	-	3.10 (1.14-8.38)	0.026
CCS 0	0.50 (0.40-0.63)	<0.001	0.50 (0.24-1.06)	0.030	-	-	0.54 (0.25-1.14)	0.046
CCS 1	0.67 (0.59-0.76)	0.115	1.07 (0.67-1.70)	0.039	-	-	-	-
CCS 2	0.70 (0.62-0.80)	0.224	1.08 (0.87-1.35)	0.476	-	-	-	-
CCS 3	0.74 (0.65-0.83)	0.332	0.91 (0.57-1.45)	0.537	-	-	-	-
CCS 4	1.46 (1.30-1.64)	<0.001	1.06 (0.67-1.66)	0.807	-	-	-	-
CCS ACS	2.07 (1.66-2.57)	<0.001	1.13 (0.28-4.54)	0.864	-	-	-	-
CAD 1VD	0.83 (0.71-0.98)	0.148	0.86 (0.56-1.34)	0.514	-	-	-	-
CAD 2VD	0.87 (0.79-0.96)	0.454	1.00 (0.78-1.27)	0.635	-	-	-	-
CAD 3VD	0.85 (0.75-0.97)	0.301	-	-	-	-	-	-
LM disease	1.23 (1.12-1.34)	<0.001	1.28 (1.01-1.64)	0.049	-	-	-	-

Previous MI	1.46 (1.34-1.59)	<0.001	1.03 (0.82-1.28)	0.812	-	-	-	-
Previous PCI	1.34 (1.23-1.47)	0.015	0.81 (0.61-1.07)	0.146	-	-	-	-
Redo surgery	1.46 (1.01-2.10)	0.043	-	-	-	-	-	-
Cardiogenic shock	4.26 (3.45-5.26)	<0.001	0.81 (0.11-5.75)	0.831	-	-	-	-
Critical preoperative state	3.95 (3.24-4.8)	<0.001	-	-	13.85 (5.7-33.64)	<0.001	-	-
IABP	4.64 (4.06-5.31)	<0.001	4.11 (3.87-4.35)	<0.001	-	-	-	-
iv. nitrates	1.46 (1.32-1.62)	<0.001	1.32 (0.97-1.78)	0.075	-	-	-	-
iv. inotropes	2.8 (2.32-3.38)	<0.001	1.26 (0.40-3.92)	0.693	-	-	-	-
Elective	0.76 (0.7-0.83)	<0.001	0.87 (0.68-1.11)	0.270	-	-	-	-
Urgent	1.19 (1.09-1.3)	<0.001	1.15 (0.90-1.48)	0.258	-	-	-	-
Emergency	2.36 (1.87-2.99)	<0.001	-	-	-	-	-	-
Salvage	4.03 (2.38-6.82)	<0.001	-	-	6.08 (2.44-15.13)	<0.001	-	-
Ablation	0.65 (0.5-0.83)	<0.001	0.86 (0.51-1.45)	0.578	0.53 (0.44-0.64)	<0.001	-	-
Aortic no-touch	0.82 (0.75-0.89)	0.020	-	-	-	-	-	-
IMA	0.72 (0.66-0.79)	<0.001	0.90 (0.68-1.20)	0.467	-	-	-	-
RIMA	0.73 (0.55-0.98)	0.036	0.47 (0.07-3.33)	0.449	-	-	-	-
BIMA	0.7 (0.51-0.96)	0.027	-	-	-	-	-	-
Pedicled IMA	1.34 (0.92-1.95)	0.129	1.22 (0.17-8.68)	0.843	-	-	-	-
Skeletonized IMA	0.83 (0.75-0.91)	<0.001	0.82 (0.63-1.05)	0.119	-	-	-	-
Radial artery	0.9 (0.69-1.18)	0.449	0.50 (0.21-1.22)	0.128	-	-	-	-
Majority arterial anastomoses	0.67 (0.6-0.73)	<0.001	0.47 (0.34-0.66)	<0.001	-	-	0.49 (0.35-0.70)	<0.001
Sequential anastomoses	0.68 (0.28-1.65)	0.315	0.82 (0.58-1.14)	0.235	-	-	-	-
Composite anastomoses	0.74 (0.31-1.78)	0.656	0.69 (0.39-1.24)	0.215	-	-	-	-
TAR	0.18 (0.1-0.34)	<0.001	-	-	-	-	-	-
Completeness of revascularization	0.96 (0.88-1.04)	0.302	1.02 (0.80-1.30)	0.859	-	-	-	-
LAAO	0.11 (0.02-0.8)	0.029	-	-	-	-	-	-
